# Cardiac vagal afferent neurotransmission in health and disease: review and knowledge gaps

**DOI:** 10.3389/fnins.2023.1192188

**Published:** 2023-06-07

**Authors:** Valerie Y. H. van Weperen, Marmar Vaseghi

**Affiliations:** ^1^Division of Cardiology, Department of Medicine, UCLA Cardiac Arrhythmia Center, Los Angeles, CA, United States; ^2^Molecular, Cellular, and Integrative Physiology Interdepartmental Program, University of California, Los Angeles, Los Angeles, CA, United States

**Keywords:** autonomic, vagus, afferent, cardiovascular disease, parasympathetic, myocardial infarction

## Abstract

The meticulous control of cardiac sympathetic and parasympathetic tone regulates all facets of cardiac function. This precise calibration of cardiac efferent innervation is dependent on sensory information that is relayed from the heart to the central nervous system. The vagus nerve, which contains vagal cardiac afferent fibers, carries sensory information to the brainstem. Vagal afferent signaling has been predominantly shown to increase parasympathetic efferent response and vagal tone. However, cardiac vagal afferent signaling appears to change after cardiac injury, though much remains unknown. Even though subsequent cardiac autonomic imbalance is characterized by sympathoexcitation and parasympathetic dysfunction, it remains unclear if, and to what extent, vagal afferent dysfunction is involved in the development of vagal withdrawal. This review aims to summarize the current understanding of cardiac vagal afferent signaling under in health and in the setting of cardiovascular disease, especially after myocardial infarction, and to highlight the knowledge gaps that remain to be addressed.

## Introduction

The ability to perceive and respond to sensory signals is essential for maintenance of physiological homeostasis, and therefore survival (Bonaz et al., 2021; Chen et al., 2021). The vagus nerve, also known as cranial nerve X, is fundamental to visceral sensory transduction and subsequent central integration. It plays an important role in interoception (Paintal, 1973; Khalsa et al., 2018; Prescott and Liberles, 2022), the process through which the nervous system can sense, interpret, and integrate signals from the body (Khalsa et al., 2018). In addition to afferent fibers, which predominate, the vagus nerve also contains parasympathetic efferent nerves in approximately an 80:20 ratio (Foley and Dubois, 1937). Named after the Latin word for “wandering,” vagal afferent fibers transmit sensory information from visceral organs of the cardio-respiratory, gastro-intestinal, endocrine, and immune systems to the central nervous system (Berthoud and Neuhuber, 2000). Upon central integration, specific efferent signals are formulated and transmitted via vagal efferent fibers to visceral organs.

Cardiac sensory information is transmitted to the brainstem via pseudounipolar vagal sensory neurons of the inferior (nodose) vagal ganglia (Berthoud and Neuhuber, 2000; Armour and Ardell, 2004). Of note, cardiac sensory information is also relayed through spinal afferent nerves, which synapse upon second-order neurons in the spinal cord (Armour and Ardell, 2004). Functionally, whereas excitation of vagal afferent neurons appears to predominantly evoke a parasympathetic efferent response (Aviado and Guevara Aviado, 2001; Campagna and Carter, 2003; Armour, 2004; Janig, 2006; Crystal and Salem, 2012), activation of the spinal afferent pathways elicit a primarily sympathetic reflex (Malliani and Montano, 2002; Wang et al., 2017). As such, these distinct afferent pathways contribute to the adjustment and fine-tuning of cardiac sympathetic and parasympathetic tone, which antagonistically and synergistically maintain cardiac homeostasis.

Upon cardiac injury, autonomic balance becomes disrupted, characterized by increased cardiac sympathetic tone and parasympathetic withdrawal (Florea and Cohn, 2014; Ardell and Armour, 2016; Van Weperen et al., 2023). These changes subsequently lead to further progression of cardiac disease, including heart failure, and increased susceptibility to ventricular arrhythmias after myocardial infarction, and therefore, sudden cardiac death (Vaseghi and Shivkumar, 2008; Ardell and Armour, 2016; Wu and Vaseghi, 2020). Recent data suggests that the decrease in parasympathetic *efferent* outflow can, at least in part, result from diminished vagal *afferent* signaling (Salavatian et al., 2022). Understanding the physiological role of cardiac vagal afferents, how cardiac injury alters their function, and how this ultimately translates to cardiac autonomic dysfunction is, therefore, of paramount importance.

## Anatomy of the vagus nerve

Peripheral terminals of cardiac vagal afferents can be found across the different layers of the atrial and ventricular myocardium and the conduction system (Foreman, 1999; Zhao et al., 2022). From the heart and mediastinum, cardiac vagal sensory neurites merge into the bilateral vagi, which span the distance between the heart and the central nervous system.

On the cranial side, just inferior to the jugular foramen or, in some species including humans, within the jugular foramen, lies the nodose, or inferior, vagal ganglion. Given the location of the nodose ganglion in humans, it is more difficult to reach surgically (Settell et al., 2020; Prescott and Liberles, 2022).

In the nodose ganglion, the somata of all vagal afferent neurons are found, interspersed with glial cells, endothelial cells, and fibroblasts (Kupari et al., 2019). Interestingly, there is no viscerotopic organization to the nodose ganglia (Akgul Caglar et al., 2021), meaning that afferent neurons of specific visceral organs are not localized to a particular region in the ganglion. Cardiac vagal afferent neurons make up approximately 5–6% of all sensory afferent neurons,(Akgul Caglar et al., 2021; Zhao et al., 2022) and given lack of a spatial distribution, there is much interest in the identification of molecular markers that are specific for the cardiac phenotype.

Superior to the nodose ganglion lies the jugular ganglion, which mainly contains vagal afferent neurons innervating the auricular and meningeal branches of the vagus. These afferent neurons/nerves are of a developmentally different origin than the vagal afferents in the nodose (neurogenic placode vs. neural crest, respectively) (Moody and Lamantia, 2015). In most species, including humans, pigs, dogs and cats, the nodose and jugular ganglia are physically separated. However, in mice and rats, the jugular and nodose ganglia are often together in one structure, interchangeably termed the nodose or jugular(-petrosal)-nodose ganglion (Kollarik et al., 2019).

Once cardiac vagal afferent nerves have passed through the jugular foramen, they project to the nuclei tractus solitarius (NTS) (Lawrence and Jarrott, 1996). In contrast to the nodose ganglion, the NTS does appear to have some viscerotopic organization; the dorsal medial part being predominantly involved in cardiovascular reflexes (Lawrence and Jarrott, 1996). From the NTS, a subset of second order neurons project to the nucleus ambiguous and dorsal motor nucleus of the vagus, from where preganglionic efferent vagal neurons project to the periphery (Standish et al., 1994). Alternatively, second order neurons can also project to higher centers of the brain, for further integration of cardiac vagal afferent information (Neff et al., 1998).

## Cardiac vagal afferent neurotransmission in health

In the healthy state, sensory information on the mechanical and chemical milieu of the heart, including the atria and ventricles, is continuously transmitted to the central nervous system through specialized mechano- and/or chemosensitive cardiac vagal neuron receptors (Hainsworth, 1991; Armour and Ardell, 2004). In the ventricles, there appears to be preferential distribution of vagal receptors on the infero-posterior left ventricular wall (Thames et al., 1978; Walker et al., 1978; Felder and Thames, 1979), whereas the highest densities of cardiac spinal afferents are found to be at the left anterior wall (Quigg et al., 1988; Quigg, 1991). Correspondingly, myocardial infarcts of the inferior left ventricular wall or application of epicardial afferent chemicals on the posterior wall elicit a primarily vagal response (Inoue and Zipes, 1987; Lombardi et al., 1996). In addition, various important reflexes are mediated through the vagus, including the Bainbridge and Bezold-Jarisch reflex (Aviado and Guevara Aviado, 2001; Campagna and Carter, 2003; Crystal and Salem, 2012), which are initiated with activation of vagal atrial or ventricular receptors, respectively. Most vagal afferent fibers seem to form flower spray or plate of puncta, parallel intramuscular arrays or end-net-like endings, based on data obtained from mice and rat aorta and atria (Cheng et al., 1997a,b; Zhao et al., 2022). Distinct from the atria, vagal afferents primarily form intramuscular array-like endings within the ventricles (Kazci et al., 2022).

Mechanosensitive cardiac vagal afferents are primarily myelinated Aδ fibers that have their endings within the epi- or endocardium (Quigg, 1991; Armour and Ardell, 2004; Shenton et al., 2021). These afferent neurons are specialized in the transduction of sensory information on myocardial mechanical deformation, generally discharging one or two impulses with each cardiac cycle. Depending on the subtype, ventricular mechanosensitive neurons are either activated by changes in ventricular filling (preload) or by increased ventricular pressure during systole (afterload) (Armour and Ardell, 2004). In the experimental setting, mechanoreceptors can be activated by increases in afterload (e.g., through aortic occlusion), or preload (e.g., by blood volume expansion) (Beaumont et al., 2013; Salavatian et al., 2022).

Important receptors for sensing visceral and cardiovascular mechanical stretch, which are also involved in the baroreflex, are the PIEZO1 and PIEZO2 channels (Zeng et al., 2018; Min et al., 2019). PIEZO2 receptors play an important role in arterial pressure sensing, as Cre-guided ablation of the glossopharyngeal and vagal PIEZO2 neurons eliminates the baroreflex (Min et al., 2019). These PIEZO2 afferent neurons appear to sense aortic pressure through their distinct terminal morphology of claw-like endings, which surround most of the aortic arch (Min et al., 2019). Recent studies have also reported a potential role of TRPC5, ASIC2, and Tentonin 3 (TMEM150c) channels in the baroreflex and sensing of changes in blood pressures in the aortic arch (Lu et al., 2009, 2020; Lau et al., 2016). TRPC5, a member of the transient receptor potential (TRP) family, is a cation channel that is activated with increases in hydrostatic pressure, hypoosmolarity, and membrane stretch (Gomis et al., 2008; Jemal et al., 2014; Shen et al., 2015). In transgenic *Trpc5* knockout mice, baroreflex-induced heart rate changes are attenuated, and blood pressure fluctuates drastically throughout the day (Lau et al., 2016). Similarly, ASIC2, a member of the acid sensing ion channel family, is another mechanosensitive channel that is expressed by aortic baroreceptor afferent neurons (Lu et al., 2009). *ASIC2* knockout mice develop hypertension and an impaired baroreflex through decreased vagal afferent sensing of aortic pressure, highlighting the role of this channel in autonomic control of blood pressure (Lu et al., 2009). Moreover, genetic deletion of tentonin 3, a cation channel that senses aortic stretch, also results in hypertension, tachycardia, and impaired baroreflex sensitivity (Lu et al., 2020). Interestingly, subsequent overexpression of tentonin 3 in these knock-out mice reverses the observed hypertension, underscoring the importance of this channel in sensing arterial pressure (Lu et al., 2020). As such, the expression of these channels can be used as a marker for mechanosensitive neurons.

Chemosensitive cardiac vagal afferents, on the other hand, are present in much greater numbers (Armour and Ardell, 2004). The majority of this population comprises of non-myelinated C fibers, which exhibit low basal activity, do not have a rhythmic discharge pattern that is synchronized with cardiac rhythm, and demonstrate a slow response upon activation (Armour and Ardell, 2004; Shenton et al., 2021).

**FIGURE 1:**
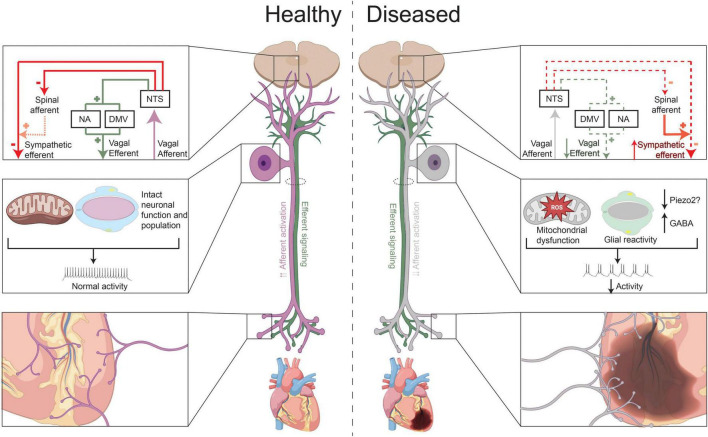
Vagal afferent transduction in health and after chronic myocardial injury. Under healthy conditions, release of neurochemicals that cause excitation of chemosensitive neurons, such as of TRPV1 + neurons, and activation of mechanosensitive afferent neurons (i.e., due to rises in ventricular or blood pressure), increase vagal efferent outflow to the heart and decrease sympathetic efferent activity. After chronic myocardial injury or infarction, vagal afferent transduction appears to be decreased. This is possibly due to increased expression and release of GABA after MI or decrease in PIEZO2 neurons (in setting of hypertension or ventricular hypertrophy), which can in turn, reduce activity of nociceptive and mechanosensitive neurons, respectively, development of metabolic dysfunction and oxidative in these neurons, and/or possibly due satellite glial cell reactivity. At the level of the heart, there is a pattern of vagal afferent denervation in the infarcted area, with vagal afferent nerve sprouting in the peri-infarct area and border zone regions. In the central nervous system, the functional decrease in vagal afferent signaling reduces vagal efferent outflow and indirectly increases cardiac sympathetic outflow.

Vagal chemosensitive afferent neurons are activated through binding of various chemicals, including bradykinin, prostaglandins, reactive oxygen species (Recordati et al., 1971; Robertson et al., 1985; Ustinova and Schultz, 1994). The majority of chemosensitive cardiac vagal afferents are transient receptor potential vanilloid-1 (TRPV1) positive C-type nociceptors (Porszasz et al., 1955; Bencsik et al., 2020; Szabados et al., 2020). This homotetrameric non-selective cation channel is best characterized for its activation through binding of capsaicin (Caterina and Julius, 2001), the main ingredient in chili peppers. However, it can be activated through a wide range of stimuli, including contact with nociceptive compounds and noxious heat (Caterina et al., 1997; Tominaga and Julius, 2000; Caterina and Julius, 2001). Moreover, several of such nociceptive compounds are produced and released upon tissue injury and ischemia, including H^+^, K^+^, bradykinin, reactive oxygen species, and prostaglandins (Nagy et al., 2004; Randhawa and Jaggi, 2015). TRPV1 activation results in Na^+^ and Ca^2+^ influx, inducing neuronal depolarization and the local release of neuropeptides, such as calcitonin gene-related peptide (CGRP) and substance P (Holzer, 1988; Maggi and Meli, 1988). Upon binding to their respective receptors, these peptides promote neurogenic inflammation (Holzer, 1988; Maggi and Meli, 1988) by inducing vasodilation, acute phase protein activation, and immune and inflammatory cell activation through paracrine signaling (Figure 1). Interestingly, whereas TRPV1 is expressed by both neurons that can release CGRP and substance P (peptidergic neurons) and non-peptidergic neurons in rats and humans (Tominaga et al., 1998; Price and Flores, 2007), TRPV1 expression is limited to peptidergic afferents in mice (Cavanaugh et al., 2011).

Experimentally, TRPV1 expressing afferent neurons can be selectively “ablated” via the administration of high doses of capsaicin (>50 mg/kg) or resiniferatoxin (RTX) (Jancso et al., 1980; Wang et al., 2015; Teofilo et al., 2019). Chemical ablation of TRPV1 afferents has been successfully tested in both the clinical and pre-clinical setting, including as a therapy chronic pain and overactive bladder (Kissin and Szallasi, 2011).

In addition to chemosensitive and mechanosensitive neurons, a substantial number of cardiac vagal afferents are multimodal in nature (Salavatian et al., 2019), meaning that they can be activated by both mechanical distortion, as well as bradykinin, nicotine, and/or veratrum alkaloids (Thoren, 1977; Baker et al., 1980; Nerdrum et al., 1986; Armour et al., 1994; Thompson et al., 2002; Armour and Ardell, 2004). These afferents, similar to the mechanosensory neurons, are primarily Aδ fibers (Armour and Ardell, 2004).

Within the NTS, vagal afferents synapse upon second-order neurons, using glutamate as their primary neurotransmitter (Armour and Ardell, 2004). Thus, most vagal afferents express a vesicular glutamate transporter. Both vesicular glutamate 1 and 2 are expressed in the nodose ganglia (Corbett et al., 2005).

In the central nervous system, glutamate can bind to both N-methyl-D-aspartate (NMDA) and non-NMDA receptors, which induce different cardiovascular effects; activation of NMDA receptors causes bradycardia, whereas binding of glutamate to non-NMDA receptors on second-order neurons results in an increase in arterial pressure (Colombari et al., 1997). Other important neurotransmitters released by cardiac vagal afferent fibers include gamma-aminobutyric acid (GABA), nitric oxide (NO), and dopamine (Granata and Woodruff, 1982; Catelli et al., 1987; Lewis et al., 1991; Machado and Bonagamba, 1992).

Gamma-aminobutyric acid is another important neurotransmitter within the NTS, which is inhibitory and expressed by approximately 20% of vagal afferent neurons in the nodose ganglia (Stoyanova, 2004). Release of GABA in the NTS has been demonstrated to increase blood pressure (Catelli et al., 1987). NO is released by vagal afferent neurons that express either the neuronal and/or endothelial isoforms of nitric oxide synthase (nNOS or eNOS) (Lawrence et al., 1997; Yamamoto et al., 2003). Injection of NO into the NTS decreases arterial pressure and heart rate (Lewis et al., 1991; Machado and Bonagamba, 1992). Lastly, dopamine release in the NTS also increases systemic blood pressure and heart rate (Granata and Woodruff, 1982).

Finally, vagal afferent neurotransmission is also likely modulated by satellite glial cells (SGC) in the nodose ganglia. SGCs have been shown to envelop the cell bodies of neurons in the peripheral sensory ganglia and interact with neurons through gap junctions and buffering of neurotransmitters (Hanani and Spray, 2020). Moreover, in the nodose ganglion glutaminergic and GABAergic signaling between neurons and SGCs has been demonstrated to increase or decrease neuronal activity, respectively (Shoji et al., 2010). However, the extent to which SGC activity modulates vagal cardiac afferent signaling remains to be elucidated.

## Cardiac vagal afferent neurotransmission in cardiovascular disease

Cardiac injury induces pathological changes in the autonomic nervous system, resulting in autonomic imbalance (Fukuda et al., 2015; Ardell and Armour, 2016; Van Weperen et al., 2023). The consequent sympathoexcitation and parasympathetic withdrawal lead to progression of heart failure and predispose to fatal ventricular arrhythmias, increasing the incidence of sudden death (Vaseghi and Shivkumar, 2008; Florea and Cohn, 2014; Shen and Zipes, 2014; Ardell and Armour, 2016; Wu and Vaseghi, 2020). It has been demonstrated that cardiac vagal efferent tone is reduced in the setting of cardiovascular disease, and that this, at least in part, is due to central vagal withdrawal manifesting as increased heart rate, altered responses to atropine, reduced heart rate variability and baroreflex sensitivity in humans and decreased vagal input to the post-ganglionic cardiac neurons of the intrinsic cardiac nervous system in large animal models of myocardial infarction (Eckberg et al., 1971; De Ferrari et al., 1991; Jouven et al., 2001; De Ferrari et al., 2007; Soliman et al., 2010; Libbus et al., 2016; Zhang et al., 2016; Vaseghi et al., 2017). Importantly, reduced vagal tone is a marker of mortality and ventricular arrhythmias (Jouven et al., 2001; De Ferrari et al., 2007; Soliman et al., 2010; Vaseghi et al., 2017). Recent data, however, suggests that these changes in parasympathetic efferent tone may at least in part, be due to reduced vagal afferent signaling (Salavatian et al., 2022).

The importance of intact vagal afferent signaling was highlighted in a study by Aydin et al. (2011) which showed that disturbed vagal afferent transduction, caused by ischemic lesions of the nodose ganglion, resulted in significant heart rhythm disturbances in all animals, and even death, in 30% of animals. The animals who died demonstrated more severe axonal degeneration of the vagus compared to the animals that survived) (Aydin et al., 2011). Moreover, under normal conditions, vagal cardiovascular afferent neural activity tonically inhibit sympathetic efferent neurotransmission through central pathways (Figure 1; Ammons et al., 1983; Ren et al., 1990; Sved et al., 2001). Impaired or decreased vagal afferent signaling can, therefore, result in increased cardiac sympathetic efferent outflow. Correspondingly, in the nucleus ambiguous and dorsal motor nucleus of the vagus, activity of cardiac vagal neurons (identified using retrograde tracers) is decreased after left ventricular hypertrophy induced heart failure, mediated by spontaneous inhibitory GABAergic neurotransmission (Cauley et al., 2015). Isolated electrical stimulation of vagal afferents (through esophageal electrical stimulation) on the other hand, decreases sympathetic and increases parasympathetic tone of the heart, as reflected by power spectral analyses (Bajwa et al., 1997).

### Myocardial infarction

Nearly 850,000 people suffer from myocardial infarction (MI) each year in the United States (Tsao et al., 2022). Clinical and pre-clinical studies have detailed the resulting autonomic imbalance, with most focused on sympathetic (efferent) signaling (Chidsey et al., 1962; Ardell and Armour, 2016). Only a few anatomical studies have evaluated the effects of (chronic) ischemia on vagal afferent signaling. Barber et al. (1985) found that upon embolization of the first or second diagonal branch of the left anterior descending coronary artery, vagal afferent signaling distal from the lesion, as identified by efferent vagal responses, were diminished. Similarly, Inoue et al. (1988) found that cardiac ischemia interrupts spinal and vagal afferent signaling, but that the attenuation in afferent signaling can be reversed by reperfusion. Given that this study allowed for reperfusion after only 15 min of ischemia, it remains unknown if reperfusion would also rescue afferent nerves after longer periods of occlusion. Kazci et al. (2022) evaluated vagal afferent remodeling in a mouse model of chronic myocardial infarction. They observed vagal afferent denervation in the infarcted area, with vagal afferent nerve sprouting in the peri-infarct area and border zone regions. This reported pattern of hyperinnervation resembles those of sympathetic efferent fibers after MI, induced by a temporal increase in neuronal growth factor (Figure 1; Fukuda et al., 2015). Finally, a study by Ieda et al. (2006) showed that neuronal growth factor could rescue loss of cardiac spinal afferents in a diabetic mouse model. It is therefore, possible that neuronal growth factor could also induces reinnervation of vagal afferent fibers, though additional data is needed.

Functionally, Salavatian et al. (2022) were one of the first to study the effects of myocardial infarction on cardiac vagal afferent signaling in a large animal model. Using *in vivo* neural recordings from the nodose ganglia of pigs with chronic myocardial infarction, they observed an increase in the *number* of nociceptive neurons, but a paradoxical decrease in *functional* nociceptive signaling (Salavatian et al., 2022). More specifically, upon application of nociceptive chemicals on the heart, they observed a predominantly inhibitory response of vagal cardiac afferents, whereas the healthy control animals exhibited an excitatory response. This functional difference was associated a significantly greater number of CGRP-positive neurons that also expressed GABA in infarcted animals (Salavatian et al., 2022). Hence, this increase in GABA could, at least in part, be responsible for mediating the observed inhibitory responses. Other studies had previously shown that GABA can be released by nociceptive neurons (Du et al., 2017). GABA has been shown to not only reduce the activity of the releasing neuron, but to also act in a paracrine fashion to inhibit the function of nearby nociceptive neurons (Hanack et al., 2015). Therefore, the increased expression of GABA in vagal afferent nociceptive neurons could result in functionally lower activity (Du et al., 2017). Notably, this study did not observe any changes in quantity or quality of mechanosensitive neurons (PIEZO2) neurons, but did find an increase in glial fibrillary acidic protein (GFAP) expression, which could indicate increased SGC reactivity (Figure 1; Salavatian et al., 2022). However, the increase in glial fibrillary acidic protein (GFAP) expression was not selective for CGRP-expressing neurons.

Similarly, the effects of MI on parasympathetic efferent responses to vagal afferent activation were studied in a transgenic mouse model, in which glutamatergic vagal afferents could be activated through optogenetic stimulation (Lokhandwala et al., 2021). In this study, a decreased vagal efferent responses to similar levels of afferent activation were observed (Lokhandwala et al., 2021). It’s possible that in addition to a potential role for GABA, vagal dysfunction could be a result of myocardial infarction induced metabolic dysfunction in the vagal ganglia, similar to what has been observed in the central nervous system of patients with heart failure and myocardial ischemia (Fiechter et al., 2019; Bai et al., 2022). In rats with chronic heart failure, decreased baroreceptor sensitivity was associated with mitochondrial dysfunction in the nodose ganglia, leading to reduced activation of voltage-gated sodium channels in these baroreceptor neurons (Tu et al., 2012). This effect could be partially restored by *in vivo* transfection of manganese superoxide dismutase, which resulted in decreased mitochondria-derived superoxide levels in the nodose ganglia (Zhang et al., 2014). Therefore, mitochondrial dysfunction and oxidative stress may play an important role in the development and progression of vagal afferent dysfunction.

Since after myocardial infarction, cardiac vagal nociceptive signaling appears to be reduced, it’s possible that a subsequent nociceptive stimulus (i.e., additional ischemia) in the setting of post-myocardial infarction autonomic remodeling will result in reduced parasympathetic responses, potentially increasing the risk of ventricular arrhythmias and mortality. Premature ventricular contractions and cardiac ischemia, for example, can activate both chemosensitive and mechanosensitive vagal afferent neurons (Recordati et al., 1971; Robertson et al., 1985; Salavatian et al., 2019). In the setting of ischemia, various substances are released that excite these cardiac afferent neurons, including bradykinin, thromboxane A2, prostaglandins, free radicals, lactic acid, and adenosine triphosphate (ATP) (Ustinova and Schultz, 1994; Pan and Chen, 2004; Kumar et al., 2019). Whereas under healthy conditions, activation of these nociceptors would increase vagal afferent neuronal firing, and reflexively, increase parasympathetic efferent outflow, reduced signaling in the setting of a second ischemic insult can (Figure 1) can potentially face decreased vagal tone and predispose to life-threatening arrhythmias.

### Hypertension

Chronic hypertension and consequent left ventricular hypertrophy are also associated with changes in cardiac autonomic remodeling. However, few studies have evaluated the role of vagal afferent neurotransmission in contributing to this sympathovagal imbalance. Huo et al. (2021) found a decrease in expression of PIEZO2 positive mechanosensitive neurons in the nodose ganglia of spontaneous hypertensive rats as well as in a hypertensive rat model. These results suggest diminished cardiac vagal afferent mechanotransduction in the setting of chronic hypertension (Figure 1).

## Knowledge gaps

Even though data on vagal afferent (dys)function remains limited, vagal sensory neurotransmission appears to play a key role in regulating parasympathetic efferent outflow to the heart and in the development of cardiac autonomic dysfunction. Several aspects of cardiac vagal afferent neurotransmission in health and disease require further investigation, and significant knowledge gaps remain. Specific and sensitive markers of cardiac vagal afferent neurons are lacking. Most studies have used TRPV1, PIEZO1, PIEZO2, CGRP, VGLUT1, and/or VGLUT2 or a combination of these markers. However, as previously mentioned, these markers are neither specific nor sensitive for cardiac-specific sensory neurons. Recent studies have combined single cell RNA sequencing combined with cardiac tracing to specifically study the transcriptomic profile of cardiac vagal afferent neurons (Kupari et al., 2019; Zhao et al., 2022). However, this has yet to lead to the identification specific cardiac afferent neuronal molecular markers.

Multi-organ or nerve tracer studies have alluded to the presence of convergent neurons with dichotomizing afferents within the peripheral autonomic ganglia, which may receive sensory information from multiple visceral organs (Pierau et al., 1984; Christianson et al., 2007; Devarajan et al., 2022; Zhao et al., 2022). In the vagal ganglia, Devarajan et al. (2022) noted a rather large proportion of cardiac and respiratory neurons to be convergent or cardiorespiratory, as delineated via differential viral and dye tracer studies in the heart and lungs, suggesting that these neurons receive inputs from both organs. It is known that cardiorespiratory integration at the level of the brainstem is critical for regulation of heart rate changes associated with breathing. Nucleus ambiguus and dorsal motor nucleus of vagus receive inhibitory inputs from the pre-Bötzinger complex (the primary respiratory rhythm generator in mammals) and pulmonary stretch receptors via the NTS (Feher, 2012; Menuet et al., 2020). Given the presence of convergent neurons in the vagal ganglia, it’s possible that some integration of these reflexes occurs even before signaling reaches the brainstem. Examples of axonal convergence and divergence are widely present in the central nervous system (Man et al., 2013). Moreover, many pathologies that are initiated within one organ (system), such as a myocardial infarction, impact multiple organs (e.g., the respiratory system). As the vagus nerve harbors afferent fibers from several visceral organs, it is possible that multi-organ interactions may at least be partially “integrated” within the nodose sensory neurons. Elucidating how vagal neurons are involved in transducing and integrating multi-organ autonomic innervation could result in a better understanding of how multi-organ pathologies interact through their common autonomic innervation.

Few studies have assessed changes in vagal afferent dysfunction after cardiac injury. As this review has highlighted, the knowledge on altered vagal afferent signaling has been mainly derived from experimental studies looking at the changes induced by myocardial infarction or hypertension. However, the role of vagal afferent dysfunction in other cardiovascular disease, such as heart failure with preserved ejection fraction, are largely overlooked. Moreover, the molecular signals and cellular adaptations that underlie reduced vagal afferent signaling, as well as therapeutic approaches that can impede or prevent these changes, remain to be elucidated. While vagal nerve stimulation had been initially put forth as a promising neuromodulatory approach in preclinical and clinical studies of heart failure, it has been met with mixed results in large randomized clinical trials (Li et al., 2004; Schwartz and De Ferrari, 2009; Sabbah et al., 2011; Hamann et al., 2013; Premchand et al., 2014; Zannad et al., 2015; Gold et al., 2016). Studies have often over looked the fact that the vagus nerve is largely made up of afferent fibers, and effects of activating these fibers electrically remains unclear. A few large animal studies have suggested that electrical stimulation of the vagus does cause afferent fiber activation that seems to paradoxically reduce cardiac vagal efferent effects, though which subtypes of fibers are leading to this inhibition are unknown (Ardell et al., 2015; Yamakawa et al., 2015). Having a better understanding of the integrated effects of afferent and efferent vagal nerve stimulation might be beneficial in further improving and targeting this neuromodulatory therapy.

Lastly, SGC dysfunction and reactivity has been demonstrated to play a significant role in the development and progression of various neuropathic diseases, including chronic pain (Hanani and Spray, 2020). Even though SGCs have been shown to be present in the nodose ganglia, the extent to which they actively modulate vagal afferent signaling remains unknown. As SGCs can release TNFα and other excitatory cytokines (Zhang et al., 2007; Song et al., 2014), it’s possible that their activation may contributed to vagal afferent dysfunction.

## Conclusion

Cardiac vagal afferent signaling is fundamental for maintenance of cardiac autonomic balance. After chronic cardiac injury, parasympathetic efferent withdrawal appears to be, at least in part, mediated through decreased cardiac vagal afferent signaling. However, compared to efferent signaling within the cardiac neuraxis, studies on parasympathetic afferent neurotransmission are still in their infancy. Additional data on the contribution of cardiac vagal afferent neurotransmission in health and in the setting of cardiovascular disease could provide key insights for the design of improved, targeted neuromodulatory therapies with reduced off-target effects.

## Author contributions

Both authors contributed to the design, literature search and review, and drafting of the manuscript.
